# Exploring the preferences of involved health professionals regarding the implementation of an online decision aid to support couples during reproductive decision-making in hereditary cancer: a mixed methods approach

**DOI:** 10.1007/s10689-019-00119-7

**Published:** 2019-01-17

**Authors:** Kelly Reumkens, Christine E. M. de Die-Smulders, Liesbeth A. D. M. van Osch

**Affiliations:** 1 Department of Clinical Genetics, Maastricht University Medical Centre+, P.O. Box 5800, 6202 AZ Maastricht, The Netherlands; 2 GROW School for Oncology and Developmental Biology, Maastricht University Medical Centre+, Maastricht, The Netherlands; 3 Department of Health Promotion, School CAPHRI, Faculty of Health, Medicine and Life Sciences, Maastricht University, Maastricht, The Netherlands

**Keywords:** Hereditary cancer, Decision aid, Prenatal diagnosis, Preimplantation genetic diagnosis, Patient participation, Implementation

## Abstract

To support persons having a genetic predisposition to cancer and their partners during reproductive decision-making, an online decision aid was developed and evaluated. To maximize the impact of the support tool, this mixed methods study aims at developing the optimal implementation strategy for the decision aid. A questionnaire to assess the critical determinants that may affect this implementation was completed by health professionals involved in oncogenetic counselling (N = 46). Subsequently, semi-structured focus groups (N = 19) and individual telephonic interviews (N = 15) were performed with a subset of health professionals. All health professionals indicated to be willing to refer couples to the decision aid, preferably at the moment of receiving the genetic test result. They agreed that the primary requirement for implementation in daily practice was ease of referring couples and preferably free online accessibility. Referral to the tool was able to be included in the standard report couples receive after consultation, thereby making the use of additional paper-based materials redundant (e.g. flyers). Furthermore, incorporating the link to the decision aid on patient organization websites was suggested. Health professionals agreed that implementation would benefit more from promoting awareness regarding the decision aid rather than the inclusion of the tool in official clinical guidelines. To foster implementation of the decision aid, the distribution of online newsletters and the designation of a contact person charged with continued implementation in each Clinical Genetic Center were suggested. Based on these preferences and recommendations, the implementation of the online decision aid will be nationally executed to optimize impact.

## Introduction

In recent decades, an increasing number of evidence-based patient decision aids have been developed to assist patients in the decision-making process of (complex) medical decisions. They have been proven effective in improving decision quality while enhancing, not replacing, the traditional process of patient counselling by practitioners [1]. However, decision aids usually do not automatically fit into routine care and are often not routinely used in daily practice [2, 3]. To optimize the impact of patient decision aids, it is therefore of great importance to focus on the active implementation after proven effectiveness [4, 5]. Significant gaps have been indicated between the number of patients eligible, the number of patients actually provided successfully with the decision support tool, and those who actually used the tool [6–8]. Although the implementation phase is considered to be a complex process, it is important to optimize this process of providing eligible patients with the tool to optimize the overall reach.

Research efforts with regard to decision support in the area of hereditary cancer are lagging behind compared to applications in non-hereditary cancer (e.g. treatment options), prenatal testing and in pregnancy care in general [1]. There have been some initiatives to support persons with a predisposition to hereditary cancer in their decision-making regarding genetic testing [9, 10] and communicating genetic test results with family members and children [11]. However, no initiatives to support couples with a predisposition to hereditary cancer in the decision-making regarding their wish to have children have been reported in the literature. The present study is part of a larger study on the development, evaluation, and implementation of an online decision aid with the aim of supporting persons having a genetic predisposition to cancer and their partners in making an informed reproductive decision [12–14]. The decision aid is meant for couples who have a high genetic risk, mostly 50%, due to autosomal dominant inheritance, of transmitting a pathogenic variant to offspring with a high risk of a future malignancy (e.g. Hereditary Breast and Ovarian Cancer, Lynch syndrome, and Familial Adenomatous Polyposis coli). Consequently, these couples face complex reproductive decisions [15–20]. Some couples may decide to refrain from having children, while others choose for donor gametes or to adopt children. Most couples, however, pursue their wish to have genetically related children [21]. Therefore, the decision aid focuses on the options to fulfil the wish for a child that is genetically related to both partners, namely; (1) natural conception without genetic testing, implying acceptance of the risk of passing on the pathogenic variant; (2) natural conception with prenatal diagnosis (PND), offering the choice to terminate the pregnancy if the fetus has the pathogenic variant [22]; (3) preimplantation genetic diagnosis (PGD), offering couples the option to obtain embryos by in vitro fertilization (IVF) and examine them for the familial pathogenic variant. Only embryos without the pathogenic variant will be transferred into the uterus [22].

The decision aid was developed as a tool to be used in the home environment and has been proven to be effective in supporting couples during their reproductive decision-making process. A reduction in mean decisional conflict and decisional self-efficacy scores, an increase in knowledge levels, levels of deliberation and improved realistic expectations were found [13, 14, 23]. This study aims at developing the optimal implementation strategy for the decision aid by investigating the preferences of health professionals involved in oncogenetic consultations in the Netherlands. Engaging these health professionals, who will be the primary sources of referral during the implementation phase, is important to understand potential barriers and find solutions on how to overcome these barriers. We used a mixed methods approach with a questionnaire, focus groups, and individual interviews.

## Methods

### Participants and recruitment

Health professionals of all Clinical Genetics Centers in the Netherlands (N = 9) were selected by a staff member of each center and were invited by email to participate in this study. All health professionals were involved in the oncogenetic counselling of couples. The email contained a short description of the goal of this study and the estimated time investment required. Inclusion criteria were experience with the counselling of persons having a genetic predisposition to cancer and their partners of reproductive age, understanding of the Dutch language, and knowledge about the existence of the decision aid and its main goals. It was allowed to forward the invitation to eligible colleagues who had not been invited yet. After permission for participation had been granted, they received a link to the online questionnaire and date proposals for a focus group or individual interview. It was allowed to complete the questionnaire without participating in an interview afterwards. The participants in a focus group or individual interview received a login code to the decision aid by email, and were asked to review the decision aid prior to the interviews.

### Content of the decision aid

An extensive explanation of the developmental process and the specific content of the decision aid is provided elsewhere [13]. Overall, the decision aid contained:

1. Information about the risk of transmitting the pathogenic variant to offspring and couples’ options to have children genetically related to both partners.
2. Treatment burden of reproductive options and the chances of different outcomes (e.g. risk of miscarriage after PND) presented in multiple suitable formats using text and videos (e.g. verbally, and through population diagrams) [12, 24].
3. A comparative summary table of important features of each option.
4. Value clarification exercises (VCE) [25]. A total of 18 statements represent values and motives considered important for reproductive decision-making [17].
5. A combined overview of both partners’ responses on the VCE, by linking login codes.
6. A question prompt sheet, providing examples of questions and requests for additional information and space for own questions.
7. Information regarding the scientific resources used to underpin the decision aids content, the development team, funding resources, and contact information.

### Instrumentation

The online questionnaire collected basic data on gender, age, job title, and participant’s work experience in counselling couples (1 = less than one year of experience, 5 = more than ten years of experience) and included a section regarding implementation of the decision aid. Firstly, an assessment was made of how detailed health professionals would like to discuss the decision aid during consultations 1 = joint use of the decision aid (i.e. reviewing and filling out the decision aid together with the couples during consultation); 2 = referral of couples to the decision aid and discussing the main results afterwards during a follow-up consultation; 3 = referral of couples to the decision aid without discussing the main results afterwards; 4 = otherwise. Secondly, nine items of the Measurement Instrument for Determinants of Innovations (MIDI) were used. The MIDI is an instrument for assessing the critical determinants that may affect implementation of innovations (e.g. decision aids). Two categories of determinants were addressed in the questionnaire: (1) determinants associated with the innovation (i.e. compatibility and relevance for client); and (2) determinants associated with the adopting person (i.e. descriptive norm, self-efficacy, awareness of content of innovation, patient satisfaction, outcome expectations, cooperation of patient, and professional obligation) [26].

A subset of the health professionals participated in a focus group or individual interview. A focus group was scheduled where three or more health professionals per Clinical Genetic Center were willing to participate. Focus groups were held at convenient workplaces for health professionals throughout the Netherlands. In the case of just one or two participating health professionals per center, individual interviews by phone were scheduled. The focus groups and interviews were conducted by one researcher (K.R.; female PhD student) who has extensive experience in conducting and analysing qualitative research and data. To direct the focus groups and individual interviews, a semi-structured topic guide was developed, based on the MIDI [26]. The content of the topic guide focused on the professional perspectives of health professionals regarding the implementation of the online decision aid within oncogenetic consultations in the Netherlands. The topic guide contained three main themes: (1) *the innovation* [i.e. perceived appropriateness of the decision aid for referral and preferences regarding availability of the decision aid (i.e. freely accessible or restricted access)]; (2) *the adopting person* (i.e. perceived personal advantages and/or disadvantages regarding the use of the decision aid by couples and best point in time for couples to review the decision aid); (3) *referral possibilities and preferences* (i.e. best point in time to refer couples to the decision aid, preferences with respect to discussing the results of the decision aid, e.g. only referral or discussing the main results, and facilitators and barriers for referring couples). The topic guide was similar for the focus groups and interviews and was pretested in an individual telephonic interview, which was included in the analyses since the interview did not result in significant changes to the topic guide. All interviews were audiotaped and the researcher took observational notes during the interviews.

### Statistical analyses

Data from the questionnaire were analyzed by means of descriptive statistics, using SPSS version 23. Qualitative data derived from the audiotaped interviews were analyzed by one researcher (K.R.). The audiotaped interviews were replayed several times and summarized to derive main themes and to explore preferences of involved health professionals regarding the implementation of the decision aid. The coding structure was developed based on the first interview by two authors (K.R. and Dr. L.v.O.), however K.R. performed coding of the remaining interviews.

## Results

### Questionnaire

#### Participants’ characteristics

A total of 46 health professionals completed the questionnaire (two males and 44 females). We were unable to calculate a response rate due to the large number of health professionals forwarding the invitation email to recruit eligible colleagues. One female participant was excluded from data analyses (dropout 2.2%) as she indicated not being aware of the existence of the decision aid. The mean age was 43.4 years (SD = 10.69). The participants were clinical geneticists (N = 23), genetic counsellors (N = 9), medical students in residency (N = 7), social workers (N = 2), physician assistants in training (N = 2), a nurse practitioner (N = 1), and a physician specialized in reproductive counselling (N = 1). Most health professionals (60.0%) had more than five years of work experience in the area of oncogenetic counselling.

The majority preferred to solely refer couples to the decision aid (68.9%), whereas some also wished to discuss the main results (24.5%). Only one counsellor was in favor of reviewing the decision aid in its full extent with couples (2.2%), and two health professionals were unsure (4.4%).

#### Determinants associated with the innovation and adopting person (MIDI)

The majority of the health professionals (38 out of 45) had reviewed the decision aid at least once (84.4%) before completing the questionnaire; the others had not done this or were yet to do this. Of these 38 health professionals, almost all thought that couples would be satisfied with the decision aid (92.1%), and found the decision aid appropriate to be used by couples (92.1%). Of the 45 health professionals who completed the questionnaire, 64.5% thought that couples would actually use the decision aid, 33.3% were unsure and 2.2% did not think so. The majority thought that it was part of their professional duties to refer couples to the decision aid (73.3%). More than half of the health professionals found the decision aid currently compatible within their daily routine clinical practice (57.8%). Almost all (91.1%) thought that they were able to refer couples to the decision aid, and 88.9% thought that at least half of their colleagues would actually refer couples to the decision aid. Almost all health professionals (93.3%) acknowledged the importance of improving informed decision-making among couples by offering them the decision aid, but just over half of the health professionals (55.5%) expected this goal to be accomplished, in contrast to 44.5% who indicated being unsure.

### Focus groups and individual interviews

A total of 34 health professionals (all females) agreed to participate in a focus group or individual interview. Three to seven health professionals (total N = 19) joined in per focus group and 15 health professionals participated in the interviews. The interviews were performed in April and May 2018. The average duration was 38 min (range 31–44) for the focus groups and 34 min (range 25–52) for the individual interviews. After three focus groups and 12 interviews, data saturation seemed to have been achieved. One additional focus group and three additional interviews were subsequently conducted in which no new or salient data were generated and data collection was closed.

#### The innovation

##### Perceived appropriateness of the decision aid for referral

All health professionals agreed that the current version of the decision aid was appropriate to provide to eligible couples and no suggestions for improvements were provided. When referring couples to the decision aid, the majority of the health professionals noticed enthusiasm among couples for using the decision aid. After using the decision aid couples provided overall positive feedback with the decision aid being pleasant in its use and helpful in improving couples’ knowledge levels.

##### Preferences regarding availability of the decision aid

All health professionals but one were in favor of a freely available tool on the Internet instead of restricted accessibility by means of unique login data distributed by health professionals. Unique login data were indicated to be a barrier for health professionals as this requirement made it complicated to refer couples to the decision aid. Furthermore, a freely available tool may make the decision aid more easily accessible for couples and other interested relatives.

#### The adopting person

##### Perceived personal advantages and/or disadvantages regarding use of the decision aid by couples

Health professionals who saw couples during follow-up consultations indicated that those couples who reviewed the decision aid were well-informed. This was indicated by almost all health professionals to be one of the key advantages of the decision aid for health professionals themselves, as it could contribute to saving time through not having to explain all elements of each reproductive option in detail during consultations. Health professionals indicated that instead of focusing merely on information provision, they have more time left to ask couples about psychological issues during reproductive decision-making and couples’ motives, consideration and values, which might lead to more interactive consultations.

##### Best point in time for couples to review the decision aid

Almost all health professionals indicated that the best point in time for couples to review the decision aid should be left to couples’ considerations. The reproductive decision-making process is complex and dynamic, and health professionals agreed that the best moment to review the decision aid differs for each couple. The emotional and psychological state of a couple plays an important role in deciding the best moment to use the decision aid.

#### Referral possibilities and preferences

##### Best point in time to refer couples to the decision aid

The way in which the genetic test result is communicated with a person differs per Clinical Genetic Center. In some centers results are explained in a consultation, whereas in other centers a letter is sent. In some centers a phone call is the usual way to communicate results. Most couples will not visit the Clinical Genetic Center for follow-up consultations after receiving the genetic test result. Follow-up consultations are only planned for persons who indicate that they are in need of additional support. Therefore, health professionals agreed that referral of eligible couples to the decision aid was best done at the time of receiving the genetic test result.

##### Preferences with respect to discussing the results of the decision aid

Although most health professionals were willing to discuss the main results of the decision aid with couples, almost all health professionals indicated that in practice no follow-up consultations are planned and thus the results of the decision aid will not be discussed with the person or couple. Furthermore, due to time restrictions within consultations, referring couples to the decision aid is most feasible. An option that seemed appropriate to all health professionals would be to refer couples to the decision aid and ask couples during follow-up consultations (only if applicable) if there are any remaining questions. For those couples who would like to discuss the results of the decision aid in more detail, facilitating a consultation with a social worker was recommended by most health professionals. Almost all health professionals mentioned that, except for referring couples to the decision aid, there should be no obligations as health professionals do not want to feel compelled to take specific actions.

##### Facilitators for and barriers to referring couples

To continue referral of couples to the decision aid, all health professionals expressed the need to keep referral of couples easy and practical within daily practice. The majority found paper-based materials (e.g. flyers and business cards) inconvenient and old-fashioned although some would appreciate having flyers available to hand out to couples. A freely accessible link to the decision aid in the standard report that counselees receive after consultation does not require too much time effort, is easy to incorporate in daily practice, and there is no need to change current work processes. Incorporating the decision aid in official clinical guidelines was not deemed necessary by virtually all health professionals. Focusing on increasing the awareness regarding the decision aid [e.g. by incorporating the decision aid on patient organization websites, on the national PGD website (http://www.PGDNederland.nl), and the national website of the information center for hereditary diseases (http://www.erfelijkheid.nl)], as well as familiarising with the tool among health professionals, was indicated to be of greater importance. According to the majority, familiarising with the tool needs some time and referrals to the decision aid will occur more frequently when health professionals start to see the actual effects of the decision aid during daily counselling. In order to secure continued implementation of the decision aid, health professionals expressed the need for someone who keeps the tool up-to-date. The distribution of online newsletters was suggested. The designation of a contact person charged with continued implementation in each Clinical Genetic Center could be helpful to foster implementation of the decision aid. Some health professionals indicated that adapting the decision aid for other hereditary conditions might also be helpful to make the decision aid more generally applicable.

## Discussion

The main goal of the present study was to explore options for the optimal implementation strategy for an online decision aid within the oncogenetic setting. We found that all health professionals indicated to be willing to refer couples to the decision aid, preferably at the moment of receiving the genetic test result. Health professionals agreed that keeping referral of couples to the decision aid easy to apply within daily practice, was the primary requirement for implementation of the decision aid. Most health professionals preferred to refer couples to the decision aid without the use of paper-based materials (e.g. flyers). Free online accessibility of the tool was preferred, with referral to the tool included in the standard report couples receive after consultation. Although health professionals agreed that the current version of the decision aid is appropriate to provide to eligible couples, just over half of the health professionals indicated in the questionnaire that the decision aid fits within their routine clinical practice. The recruitment of couples for the effect study [14] occurred with flyers including unique login data for the decision aid. The experience of health professionals during this recruitment period may have contributed to the strong preference for the use of a freely accessible decision aid, as many participants indicated that they found the use of paper-based materials (e.g. flyers) inconvenient. Health professionals indicated login data and flyers to be important barriers for the implementation of the decision aid. We therefore expect that the planned adaptations for the implementation of the decision aid will increase health professionals’ confidence that referral fits within their routine clinical practice. Furthermore, incorporating the link to the decision aid on patient organization websites was suggested. Health professionals agreed that implementation would benefit more from promoting awareness regarding the decision aid rather than the inclusion of the tool in official clinical guidelines. Suggestions for increasing awareness and foster implementation of the decision aid included the distribution of online newsletters, the designation of a contact person charged with continued implementation in each Clinical Genetic Center, as well as a party responsible for updating the tool.

To accomplish optimal implementation of the decision aid, the decision aid needs to be adapted into a freely accessible tool. The tool would then be provided to couples at the moment of receiving the genetic test result through the inclusion of information about the decision aid in the standard report couples receive after consultation. In one of our previous studies, couples agreed with health professionals that the best time for implementing the decision aid would be between the moment of receiving a positive genetic test result and the follow-up consultation (if applicable) [12]. Furthermore, inclusion of information about the decision aid in the standard report couples receive after the consultation was also suggested by couples themselves [12]. Further, patient organizations need to be approached to further explore their willingness to incorporate the decision aid on their websites to increase awareness of the tool. Lastly, frequent updating of decision aids is essential due to new evidence becoming available. In the oncogenetic setting, developments in genetic testing and reproductive technologies are expected to progress rapidly. Development and evaluation of the decision aid were performed with the help of a large research- and steering group that is also actively involved in the continued development and adaptation of the decision aid. In order to ensure the decision aid being up-to-date, negotiations with patient organizations and national organizations tasked with public information provision regarding hereditary diseases will be initiated. Based on the preferences and recommendations investigated in this study, the implementation of the online decision aid will be nationally executed to optimize impact. As one of the main outcomes of this study was to adapt the decision aid into a freely accessible tool, a broader range of possible intermediaries (e.g. general practitioners, oncologists, and gynaecologists) may be considered to add other valuable insights.

Some limitations should be mentioned. First, the number of participants within the focus groups was limited and this may have restricted interaction and discussion between participants. Secondly, although the coding structure was developed based on the first interview by two authors (K.R. and L.v.O.), the coding of the remaining interviews was performed by one author only (K.R.) which may decrease the validity of our study. Lastly, transcripts were not returned to participants. Returning transcripts to participants has both pros (e.g. strengthens the trustworthiness of the findings) and cons (e.g. alter the interpretation of the original data set), and we decided to not made use of member-checking of our data.

## Conclusion

This study provides insights into preferences and recommendations of health professionals regarding the implementation of the online decision aid. These recommendations will guide the development of the optimal implementation strategy for the decision aid, which will be nationally executed to optimize impact of the decision aid.
